# Coexistence of splenic marginal zone lymphoma with hepatocellular carcinoma: a case report

**DOI:** 10.1186/1746-1596-2-5

**Published:** 2007-02-06

**Authors:** Shu-Hui Zhang, Ai-Min Xu, Jian-Ming Zheng, Miao-Xia He

**Affiliations:** 1 Department of Pathology, Yueyang Hospital of Integrated Traditional Chinese and Western Medicine, Shanghai University of Traditional Chinese Medicine, Shanghai 200437, China; 2 Department of Radiology, Eastern Hepatobiliary Surgery hospital, Second Military Medical University, Shanghai 200438, China; 3 Department of Pathology, Changhai Hospital, Second Military Medical University, Shanghai 200433, China

## Abstract

**Background:**

Coexistence of splenic marginal zone lymphoma with hepatocellular carcinoma is rare. Although some reports have suggested the possible pathogenic role of HBV, HCV, chronic and persistent antigenic stimulation in lymphoma, their role in causing lymphomas is still unclear.

**Case presentation:**

We describe a hepatocellular carcinoma with concomitant splenic marginal zone lymphoma in a 64-year-old Chinese man with cirrhosis. Serum hepatitis B virus surface antigen was positive and antihepatitis C virus antibody was negative. The resected liver mass measuring 4 × 3 × 3 cm was grey and soft with a small area of bleeding, necrosis and intact capsule. Cut surface of the spleen was red-purple and had a diffuse reticulonodular appearance indicative of prominent white pulp. On histologic sections, the liver mass was well and moderately differentiated hepatocellular carcinoma, and the splenic tumor was a specific low-grade small B-cell lymphoma. Immunohistochemical staining and gene rearrangement studies supported that the splenic tumor represents a clonal B-cell lymphoma. Therefore, the diagnosis of SMZL was made from the splenic specimen.

**Conclusion:**

To our knowledge, this is the second case report describing coexistence of hepatocellular carcinoma and splenic marginal zone lymphoma in the course of chronic HBV infection. However, we cannot assert at present that hepatitis B virus is directly involved in splenic lymphomagenesis until more information is collected from more cases in the future.

## Background

The frequency of primary lymphoma of the spleen is relatively rare, although malignant lymphoma commonly involves the spleen [1]. Splenic marginal zone lymphoma (SMZL) is a distinctive and well-characterized B-cell neoplasm that involves the spleen and various organs [2]. Although SMZL is accepted as an entity in the World Health Organization (WHO) classification [3], its histogenesis remains unclear. There are several reports of simultaneous occurrence of additional cancers and SMZL [4-6]. To our knowledge, only one reported patient who had coexistent SMZL with hepatocellular carcinoma (HCC) [4]. Here, we present a case report of SMZL with coexistent HCC.

## Case presentation

### Clinical history

A 64-year-old man was diagnosed 6 years earlier with cirrhosis secondary to chronic hepatitis B. He was noted to have splenomegaly in a routine follow up. Laboratory data were as follows: aspartate aminotransferase, 126 U/litre (normal, < 40); alanine aminotransferase, 66 U/litre (normal, < 36); alkaline phosphatase, 123 U/litre (normal, < 96); and γ-glutamyltransferase, 178 IU/litre (normal, < 96). Serum fetoprotein was 256 μg/litre (normal, < 20). Serum hepatitis B virus surface antigen was positive and antihepatitis C virus antibody was negative.

**Figure 1:**
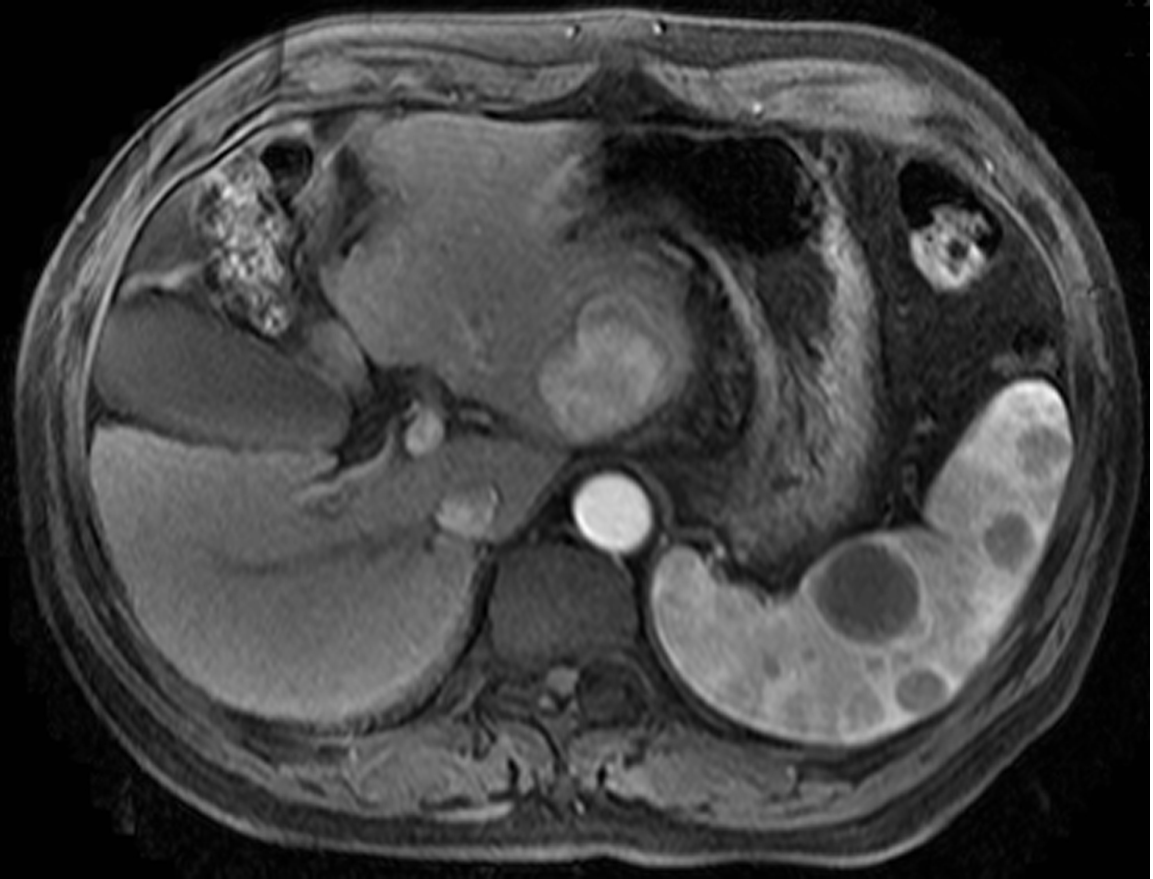
Magnetic resonance imaging showed the tumor in the left lateral segment of the liver, and a multiple, small-to-moderate nodules in the enlarged spleen.

Magnetic resonance imaging of the abdomen showed an approximately 3.5 cm in diameter with a heterogeneous, hypervascular enhancing mass in the left lateral segment of the liver. A multiple, small-to-moderate nodules composed of a low-density area throughout the parenchyma was present in the enlarged spleen. There were no tumor thrombi within the portal veins and lymphadenopathy (Figure 1).

Positron emission tomography findings revealed the ringed dense uptake of 18F-fluorodeoxyglucose in the spleen. It was not absorbed in the lymph nodes of the splenic hilus and behind the pancreatic head; there were no abnormal hot areas in the liver.

After the liver tumor and spleen were resected, the serum fetoprotein concentrations returned to within the normal range. However, the patient who was treated symptomatically and not treated with chemotherapy due to poor general condition died 6 months after surgical resection.

### Macroscopic and microscopic findings

Grossly, the resected liver tissue measured 10 × 7 × 5 cm. There was a well circumscribed tumor measuring 4 × 3 × 3 cm. The mass was grey and soft with a small area of bleeding, necrosis and intact capsule. The non-tumorous portion showed obvious cirrhotic nodularity. The resected spleen measured 25 × 17 × 7.5 cm and weighed 1750 g. The outer capsule was smooth, glistening, and intact. Cut surface of the spleen was red-purple and had a diffuse reticulonodular appearance indicative of prominent white pulp. Splenic hilar and mesenteric lymph nodes were not enlarged obviously.

**Figure 2:**
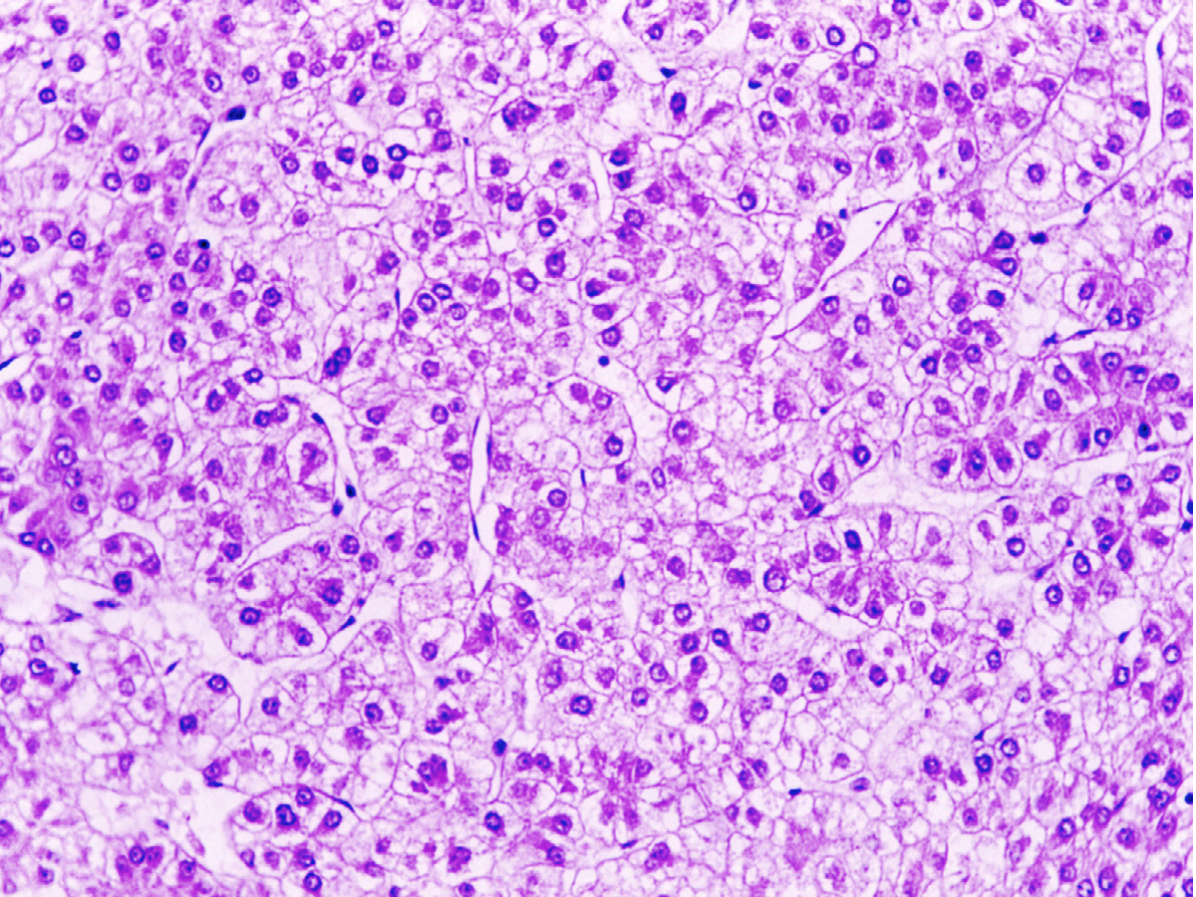
The liver tumor showed a classic hepatocellular carcinoma arranged in trabecular and acinar patterns. H&E, original magnification, ×200.

**Figure 3:**
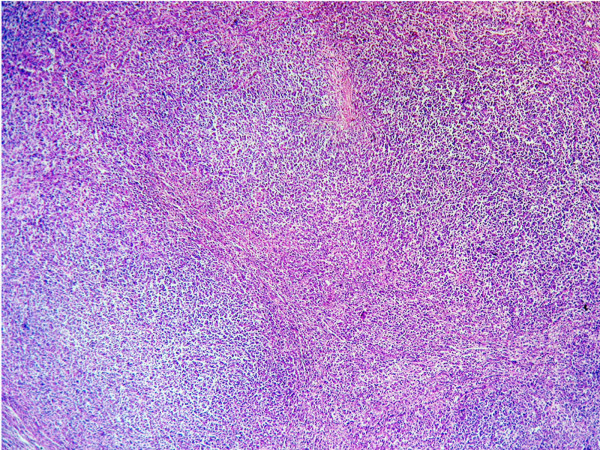
Morphology of Splenic marginal zone lymphoma. Characteristic micronodular pattern in the SMZL centred in the white pulp, with variable red pulp infiltration (H&E, original magnification ×100).

**Figure 4:**
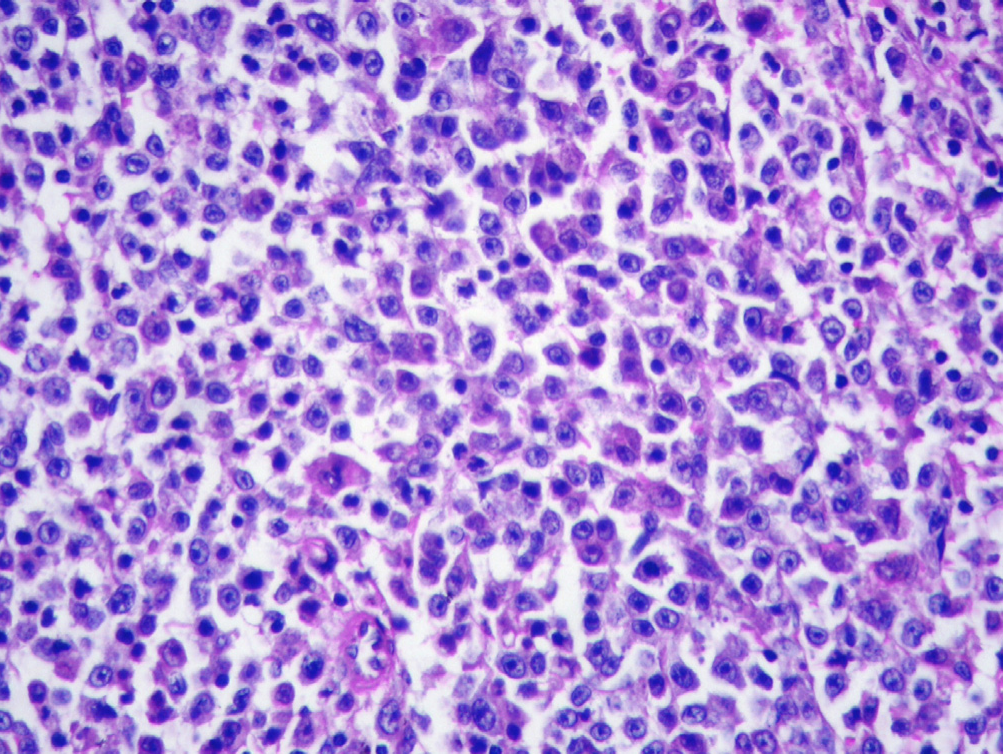
Morphology of Splenic marginal zone lymphoma. Small lymphocytes, marginal zone cells and cells resembling monocytoid cells (H&E, original magnification ×400).

On histologic sections, the tumor cells were growing in cords of variable thickness, which were separated by sinusoid-like blood spaces in the liver mass. Diffuse fatty change of tumor cells was found in small area (Figure 2). The non-tumour part showed cirrhotic change. The tumor in the spleen was characterised by a micronodular lymphoid infiltrate located in white pulp, with variable red pulp infiltration, marginal zone differentiation and follicular replacement by neoplastic cells (Figure 3). The white pulp tumoral nodules were composed of an inner central zone of small lymphocytes, located in the mantle zone and replacing the germinal center, and a peripheral zone of medium-sized cells with clear cytoplasm and scattered blasts (Figure 4), the marginal zone component. Splenic hilar and mesenteric lymph nodes were not effaced with intact capsules, sinuses, and germinal centers.

**Figure 5:**
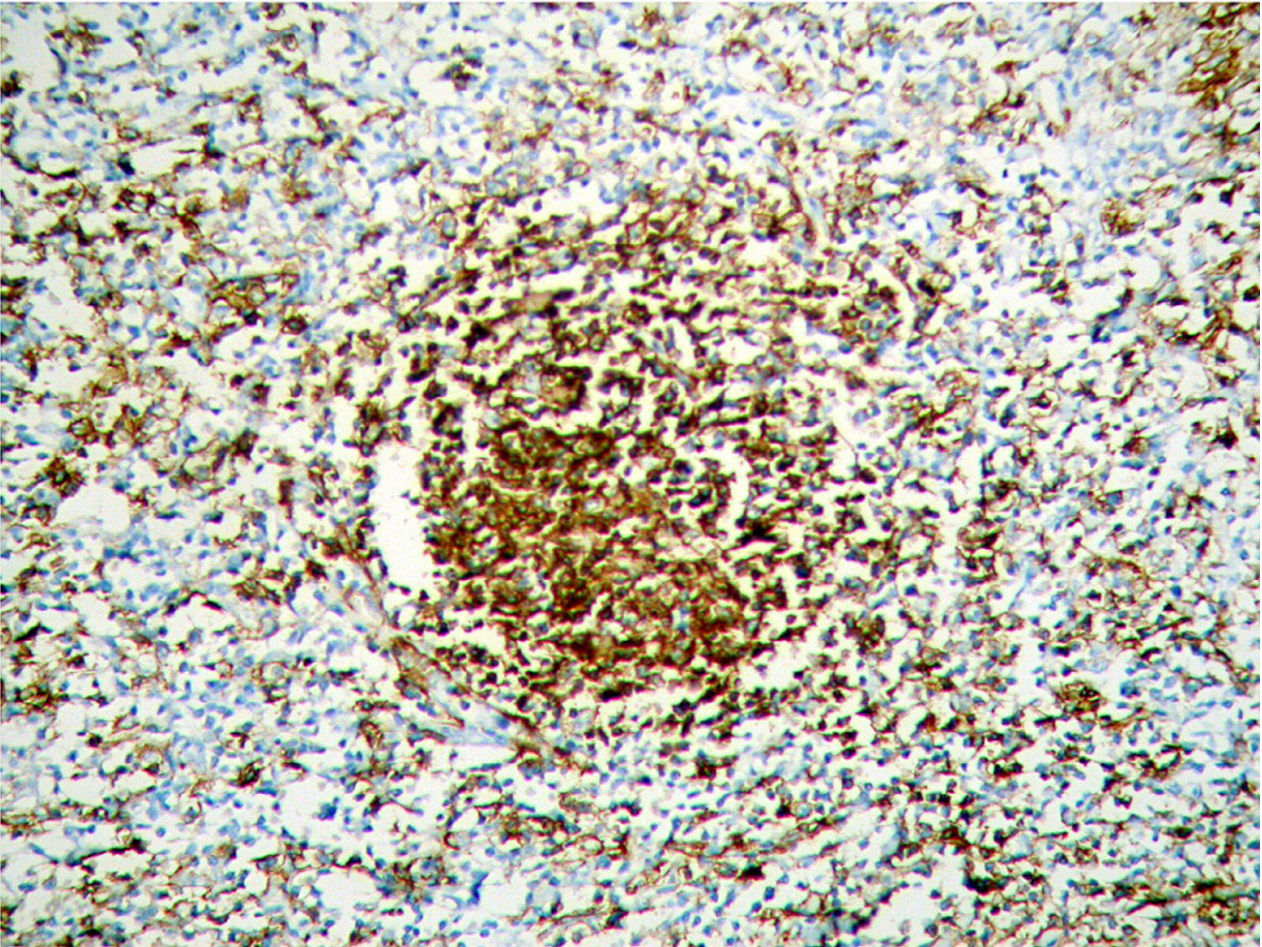
Morphology of Splenic marginal zone lymphoma. Tumor cells express CD79a (EnVision Plus, original magnification ×200).

Immunohistochemical staining of the neoplastic cells showed positivity for CD20, CD79α (Figure 5), Pax-5, bcl-2 and lacked co-expression of cyclin D, CD30, CD3, CD5, bcl-6, bcl-10, CD68, and cytokeratin, indicating that the tumor was B-cell lymphoma. The proliferative index is low, and Ki67 staining showed a distinctive annular pattern, outlining the presence of an increased growth fraction in the germinal center and marginal zone.

**Figure 6:**
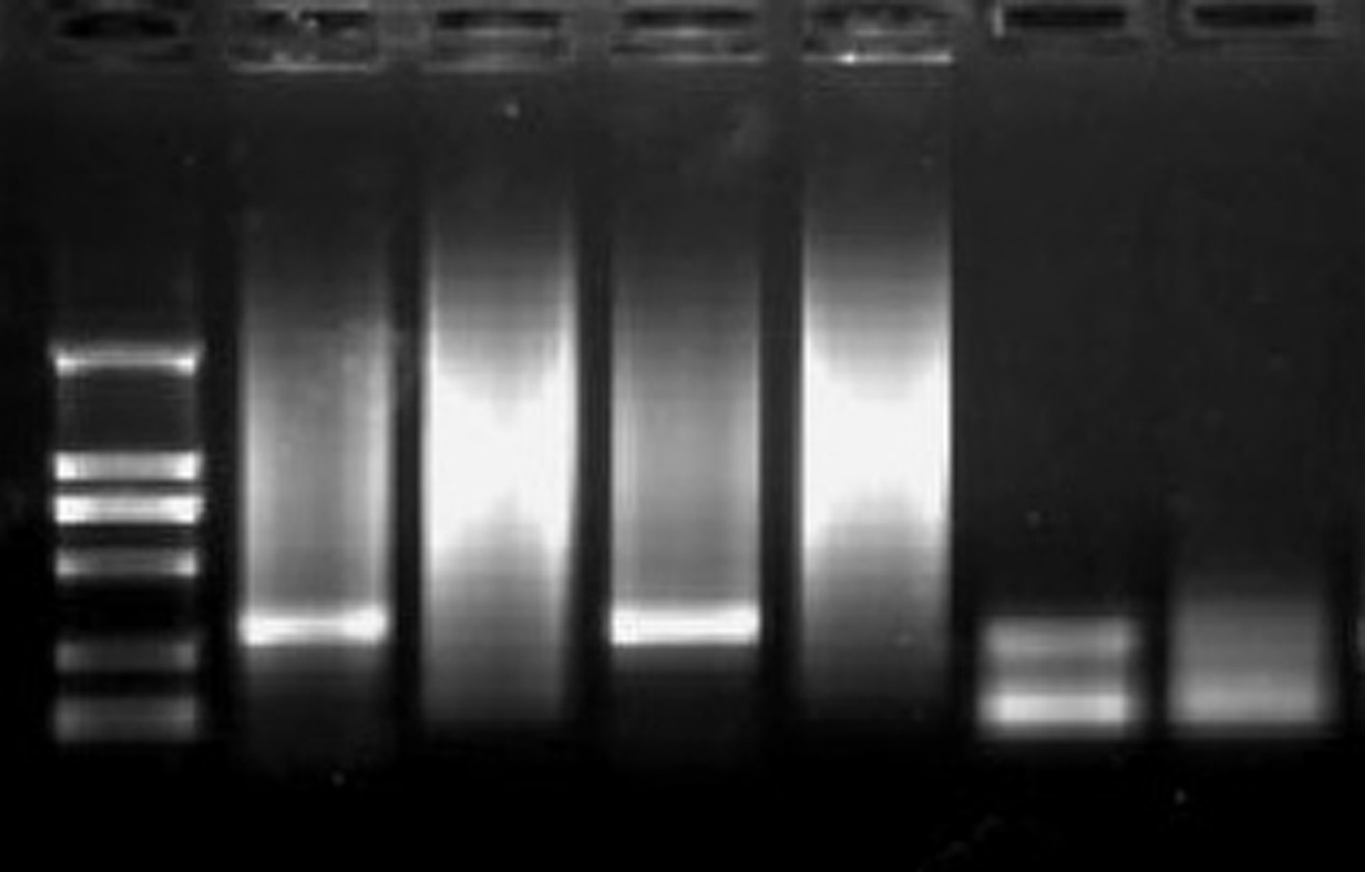
Immunoglobulin heavy-chain gene rearrangement by polymerase chain reaction. Lane M, Marker. Lane 1, FR2, diffuse large B-cell lesion lymphoma used as positive control. Lane 2, FR2, chronic tonsillitis used as negative control. Lane 3, FR2, sample collected from splenic marginal zone lymphoma showing monoclonal pattern. Lane 4, Joining region (JH), sample collected from splenic marginal zone lymphoma showing a smear. Lane 5, JH, anaplastic large cell lymphoma used as positive control. Lane 6, JH, chronic tonsillitis used as negative control.

Amplification of Immunoglobulin heavy chain genes was performed by semi-nested PCR, using primers directed to the framework 2 (FR-2) region and to the joining region (JH) as described previously [7,8] Gene rearrangement studies also supported a clonal B-cell process with heavy chain rearrangement (Figure 6).

Therefore, the liver mass was well and moderately differentiated HCC, the diagnosis of SMZL was made from the splenic specimen. The final pathological diagnosis was coexistent SMZL with HCC in the course of chronic HBV infection.

## Discussion

Coexistence of splenic lymphoma with additional cancers is rare. Iannitto et al [4] reported that twelve additional cancers in a series of 129 patients consecutively diagnosed with SMZL in three Italian centers. To our knowledge, this is the second case report describing coexistence of HCC and SMZL [4]. The B-cell nature of tumor cells in SMZL is well known [2]. The neoplastic cells are medium sized with roundish or slightly irregular nucleus, clumped chromatin, frequent small nucleolus, and a moderate amount of cytoplasm with distinct borders sometimes of villous appearance. Positivity for CD20, CD79a, Pax-5/BSAP, IgM, and bcl2 are constantly observed. T-cell antigens are always negative. Immunoglobulin heavy and light chain genes are rearranged. Differential diagnosis with lymphoplasmacytic lymphoma is more controversial, since SMZL may show plasmacytic differentiation and serum monoclonal paraproteinaemia. Absence of CD10 and bcl-6 staining are useful for excluding follicular lymphoma, and staining of cyclin D1 is helpful for excluding mantle cell lymphoma.

Chronic hepatitis B virus (HBV) or hepatitis C virus (HCV) infection is believed to play important roles in hepatocarcinogenesis. As for primary lymphoma, its histogenesis remains obscure. Recently, some reports have suggested the possible pathogenic role of HBV and HCV [9-13]. It has been reported that chronic liver disease accompanied primary lymphoma of the spleen in 14 (14.3%) of 98 cases [6]. Other models which may drive and sustain malignant transformation include chronic and persistent antigenic stimulation associated with inflammation or autoimmune disease in lymphomagenesis. Antigen stimulation leads to a polyclonal T-cell response promoting a B-cell monoclonal proliferation. Eradication of the bacteria can lead to pathologic lymphoma regression in many cases [2,5,10]. However, their role in causing lymphomas is still unclear. Therefore, we cannot assert at present that hepatitis B virus is directly involved in splenic lymphomagenesis until more information is collected from more cases in the future.

## Abbreviations

HBV, hepatitis B virus; HCC, hepatocellular carcinoma; HCV, hepatitis C virus; PCR, polymerase chain reaction; SMZL, splenic marginal zone lymphoma;

## Competing interests

The author(s) declare that they have no competing interests.

## Authors' contributions

S-H Z carried out routine and special histochemical stains. S-H Z, A-M X, J-M Z and M-X H participated equally in the design of the report and in drafting the manuscript. All authors read and approved the final manuscript.
